# Evaluation of statistical regression models in predicting factors influencing HBV and HIV among female sex workers in Ghana: A Bio-behavioural survey

**DOI:** 10.1371/journal.pone.0332152

**Published:** 2025-09-12

**Authors:** Julius Adjei-Roger, Seth Afagbedzi, Ernest Tei-Maya, Chris Guure

**Affiliations:** 1 Department of Biostatistics, School of Public Health, University of Ghana, Legon-Accra. Ghana; 2 Department of Biostatistics, School of Public Health, University of Ghana, Legon-Accra. Ghana; 3 Department of Population, Family and Reproductive Health, School of Public Health, University of Ghana, Legon-Accra. Ghana; 4 Department of Biostatistics, School of Public Health, University of Ghana, Legon-Accra. Ghana; HIV/STI Surveillance Research Center and WHO Collaborating Center for HIV Surveillance, Institute for Future Studies in Health, Kerman University of Medical Sciences, IRANISLAMIC REPUBLIC OF

## Abstract

**Purpose:**

This study aims to evaluate the effectiveness of parametric statistical methods—specifically logistic regression, Poisson regression, and Cox proportional hazards models—in identifying factors influencing Hepatitis B Virus (HBV) and Human Immunodeficiency Virus (HIV) infections among female sex workers (FSWs) in Ghana. The primary focus is to assess infection prevalence and determine how well the Cox model identifies significant predictors.

**Methods:**

A cross-sectional survey was conducted, recruiting 7,000 female sex workers (FSWs), with 5,990 completing both biological sampling and structured interviews, 5,052 for HBV and 5,426 for HIV variables. Time-location sampling ensured a representative sample. The prevalence of HBV and HIV was calculated, and a Cox proportional hazards model was employed to identify key risk factors. Hazard ratios (HRs) and p-values were used to evaluate the strength and significance of these associations.

**Result:**

The prevalence of HBV among FSWs was found to be 6.53% (95% CI: 6.08%–7.01%), while the prevalence of HIV was 4.53% (95% CI: 3.46%–5.92%). Significant predictors for HBV included alcohol consumption during sex (HR = 1.34, p = 0.042) and avoidance of healthcare due to stigma (HR = 1.64, p = 0.023). For HIV, older age was a significant risk factor, with hazard ratios of 1.60 (p = 0.007) for individuals aged 25–35 and 2.20 (p = 0.001) for those over 35 years old. Education appeared to be a protective factor: secondary education reduced HIV risk by 67% (HR = 0.33, p < 0.001), and higher education reduced risk by 60% (HR = 0.40, p = 0.019). The Cox model outperformed both logistic and Poisson regression in its ability to discriminate between risk factors and predict infection outcomes.

**Conclusions:**

The Cox proportional hazards model proved highly effective in identifying the key risk factors for both HBV and HIV. Behavioral factors like alcohol use, social determinants such as stigma, and demographic variables such as age and education played significant roles in influencing infection risks. These findings highlight the need for tailored public health interventions that address alcohol-related behaviors, reduce stigma, and improve health literacy among FSWs.

## Background

Hepatitis B (HBV) and Human Immunodeficiency Virus (HIV) are major public health concerns, especially among vulnerable populations like female sex workers (FSWs). This study focuses on HBV and HIV due to their high prevalence and significant health impacts among FSWs, particularly in sub-Saharan Africa, including Ghana. While Hepatitis C Virus (HCV) is also sexually transmitted, it was excluded from this analysis due to resource constraints and the prioritization of HBV and HIV.

Hepatitis B is a viral infection that primarily affects the liver, and as of 2019, the World Health Organization (WHO) reported approximately 296 million people living with chronic HBV infection globally [1]. The prevalence of HBV varies across regions, with the Western Pacific and African regions showing the highest rates [2]. Among female sex workers in West Africa, the risk of HBV infection is particularly elevated, with prevalence rates reaching as high as 18.2% in Burkina Faso [3]. In Ghana specifically, HBV-HIV co-infection prevalence among HIV–positive individuals is estimated at 8.8% [4]. These elevated rates highlight the need for targeted interventions, such as vaccination and screening programs among FSWs.

Similarly, HIV, which attacks the immune system and can lead to acquired immunodeficiency syndrome (AIDS) if untreated, is also prevalent among FSWs. [5] that approximately 37.7 million people were living with HIV worldwide, with sub-Saharan Africa bearing the heaviest burden [5]. In Ghana, HIV prevalence among FSWs is reported at 4.67%, with older adults (>25 years) accounting for 70% of cases [6]. HIV prevalence among FSWs has been reported at 7.5%, with studies emphasizing the need for comprehensive interventions that address both the social and structural factors contributing to FSWs’ vulnerability [7]. Key risk factors include inconsistent condom use and intravenous drug use, which facilitate the spread of HIV.

One of the most concerning aspects of HIV and HBV is the risk of co-infection, where individuals are infected with both viruses. Co-infection exacerbates health complications, particularly liver disease progression. Among FSWs, studies have found a 1% co-infection rate, signaling the vulnerability of this group to multiple infections and the critical need for enhanced prevention strategies [8]. For instance, a study in Brazil found a low co-infection rate of 0.05%, which further emphasizes the need for ongoing research and more focused prevention and treatment efforts for FSWs [9].

Sexual contact is the primary mode of transmission for both HBV and HIV, and female sex workers are at increased risk due to frequent exposure to unprotected sexual encounters. The WHO identifies over 30 pathogens transmitted through sexual contact, with HBV and HIV being two of the most significant and incurable sexually transmitted infections (STIs) [1]. Female sex workers, particularly in regions like sub-Saharan Africa and parts of Asia, are often faced with limited access to healthcare, high levels of stigma, and barriers to preventive measures such as vaccinations and antiretroviral therapy. These factors compound their vulnerability, making it essential to develop targeted public health strategies that address both the individual and structural challenges they face [10,11].

In addition to understanding the prevalence and factors for HBV and HIV, identifying the social determinants that exacerbate the risk of these infections is crucial for developing effective interventions. Factors such as poverty, substance use, and violence are common among FSWs and significantly increase their likelihood of contracting these infections [12,13]. Therefore, public health strategies must go beyond medical interventions and address the broader social issues that affect FSWs.

Parametric statistical methods play a vital role in epidemiological research, especially in identifying and analyzing the factors that influence the prevalence of diseases like HBV and HIV. These methods, including logistic regression, Poisson regression, and Cox proportional hazards models, allow researchers to model various health outcomes and identify significant predictors. In the context of HBV and HIV among FSWs, parametric methods can help identify risk factors such as sexual practices, substance use, and socio-economic status. However, these methods rely on certain assumptions, such as the normality of data distribution or proportionality in hazard models. Ensuring these assumptions are met is critical for drawing valid and reliable conclusions from the data [14–16].

The significance of this study lies not only in improving the theoretical understanding of the relationship between statistical methods and epidemiological outcomes but also in enhancing public health efforts targeting FSWs. By evaluating different statistical models and identifying the most effective approach for predicting HBV and HIV infection, this research aims to provide valuable insights for public health practitioners. The study will also contribute to the broader body of knowledge on the epidemiological determinants of these infections among FSWs, offering a nuanced understanding of their unique health challenges.

Developing a predictive model for HBV and HIV among FSWs involves the use of multiple parametric statistical techniques to capture the full range of data characteristics. Techniques like logistic regression can model binary outcomes, while Cox regression is suited for time-dependent factors. These models can be compared using metrics such as the Akaike Information Criterion (AIC) and Bayesian Information Criterion (BIC), which assess the balance between model fit and complexity [17]. Additionally, validation techniques such as k-fold cross-validation and bootstrapping methods can be used to ensure the robustness and stability of the model, preventing overfitting and enhancing its predictive accuracy [18,19].

The implications of this research extend to improving public health strategies and resource allocation for HIV and HBV prevention among FSWs. By identifying the key factors for these infections and evaluating the effectiveness of different statistical methods, this research will guide policymakers and health organizations in implementing more effective interventions. Moreover, the findings will contribute to the development of health policies that address the social determinants of health affecting FSWs, such as stigma, discrimination, and access to healthcare. By providing evidence-based insights into the factors influencing HBV and HIV infection, this study aims to support the creation of tailored interventions that can reduce the burden of these diseases among FSWs and improve their overall health outcomes.

## Methodology

### Study approach

A pre-survey assessment was conducted to ensure the robustness and relevance of data collection for evaluating HBV and HIV risk factors among female sex workers (FSWs) in Ghana. This phase involved consultations with national and regional stakeholders, including civil society organizations (CSOs) working with FSWs and representatives from the Ghana Police Service, to identify operational venues, peak times, and logistical challenges. These consultations informed the development of a comprehensive sampling frame, following established guidelines for bio-behavioural surveys among key populations [20].

### Study design, population, and setting

This cross-sectional bio-behavioural survey employed Time Location Sampling (TLS), also known as venue-day-time (VDT) sampling, to obtain a representative sample of female sex workers (FSWs) across all 16 regions of Ghana: Greater Accra, Ashanti, Western, Western North, Central, Eastern, Volta, Oti, Bono, Ahafo, Bono East, Northern, Savannah, North East, Upper East, and Upper West. TLS was appropriate due to its suitability for sampling hard-to-reach populations like FSWs, where attendance at venues varies by day and time [21,22].

Before implementation, a comprehensive mapping exercise was conducted to identify all known venues where FSWs congregate. This included recording peak days, hours of activity, and estimated FSW attendance at each site. A sampling frame was developed for each region, listing all eligible venues. From this frame, venues were randomly selected without replacement. All FSWs present at selected venues during specified dates and times were approached for participation [21,22].

Eligibility criteria defined FSWs as females aged 16 years or older who reported engaging in vaginal, anal, or oral sex in exchange for money or goods with someone other than a regular partner within the past six months, in line with the National HIV and AIDS Strategic Plan 2016–2020 [23]. Data collection occurred from 17 February to 24 March 2020, yielding a final sample size of 7,000 FSWs.

### Sample size and data completeness

A total of 7,000 female sex workers (FSWs) were recruited for this bio-behavioural survey using the Time Location Sampling (TLS) approach across all 16 regions of Ghana, as described in the Study Design, Population, and Setting subsection. Of these, 5,990 participants completed both the biological sampling (HBV and HIV testing) and the behavioural structured interviews, which included questions on male condom use by clients, alcohol consumption, and other risk factors. The analyses for HBV and HIV outcomes were based on subsets of this sample due to missing data for certain variables. Specifically, the HBV analysis included 5,052 participants, and the HIV analysis included 5,426 participants, reflecting cases with complete data for the respective outcome variables and associated predictors. Missing data primarily resulted from incomplete responses to behavioural questions (e.g., male condom use, alcohol consumption) or biological test results that were inconclusive or not recorded. These exclusions ensured the reliability of the statistical analyses, including the Cox proportional hazards model, by using only complete cases, though they reduced the sample size for each outcome variable. Sampling weights, as described in the Statistical Analysis section, were applied to adjust for the complex survey design and maintain representativeness of the national FSW population.

### HIV and HBV testing procedures

After informed consent and pre-test counselling, biological samples were collected and tested following national guidelines and study-specific protocols.

HIV testing was conducted onsite using the First Response HIV 1 + 2/Syphilis Combo Rapid Test, which detects antibodies to HIV types 1 and 2. All reactive samples were further screened using the OraQuick HIV-1/2 test. Participants reactive on both tests were classified as HIV–positive. To ensure diagnostic accuracy, all HIV–positive samples and 10% of randomly selected HIV–negative samples were sent to the Noguchi Memorial Institute for Medical Research (NMIMR) for confirmatory testing using the INNO-LIA HIV I/II Score assay. Additionally, HIV–positive individuals underwent viral load testing at NMIMR using the Cepheid GeneXpert HIV-1 cartridge.

HBV testing was performed using the First Response HBsAg Card Test to detect hepatitis B surface antigen (HBsAg) in blood samples. All HBsAg-positive cases and 10% of randomly selected negatives were retested with the Alere Determine HBsAg Test for quality assurance. Detection of HBsAg confirmed active HBV infection, whether acute or chronic.

It is important to note that rapid antibody tests for HIV and antigen-based tests for HBV may not detect recent infections acquired during the window period, the interval between exposure and when markers become detectable. This limitation means some acute infections may have gone undetected. All test results were provided onsite, and participants with positive diagnoses were referred for treatment and care through local partner NGOs.

#### Target populations.

The operational definition of FSWs was kept the same and aligned with the National HIV and AIDS Strategic Plan 2016–2020 of the Ghana AIDS Commission and the M&E/Surveillance Working Group. An FSW was defined as any female aged 16 years (i.e., the age for consensual sex in Ghana) or older who reported having exchanged sexual acts (e.g., vaginal, anal and/or oral sex) in the last 6 months with someone other than her established partner for something of value (money and material items) that would otherwise not be extended to them by their sexual partners.

#### Ethical considerations.

Ethical approval was obtained from the University of Ghana Noguchi Memorial Institute for Medical Research Institutional Review Board (CPN 083/18–19), the Ghana Health Service Ethics Review Committee (GHS-ERC 002/05/19) and the Population Council Institutional Review Board (Protocol 891), New York, USA. All participants in this study provided written informed consent. The study was voluntary, and the consenting process was explained to participants so they would willingly decide to participate. Participants had the flexibility to withdraw at any time during the survey process.

## Statistical analysis

### Sampling weights

To obtain nationally representative estimates and account for the complex design of time-location sampling (TLS), sampling weights were calculated following the University of California San Francisco’s TLS guidance for bio-behavioural surveys [21,24]. Additional details of the weighting approach are provided by Guure and colleagues [25].

Weights were computed as the inverse of the probability that a female sex worker (FSW) was sampled, adjusting for the proportion of venues sampled in each region. Specifically, the national weight combined the probability of an FSW being interviewed at a selected venue, estimated as the proportion of interviewed FSWs relative to the total counted at the venue, and the probability of that venue being selected in the region, based on the regional sampling fraction.

These weights were applied to all analyses, including descriptive statistics, logistic regression, modified Poisson regression, and Cox regression, to ensure valid population-level inferences. The regression models were implemented using the svy command in Stata, which adjusts for the complex sampling design and produces robust standard errors. This approach ensures that the estimated associations and corresponding confidence intervals accurately reflect the national distribution of female sex workers in Ghana.

### Study variables

#### Outcome variables.

This study focuses on two main outcome variables: HIV infection and hepatitis B infection among female sex workers. HIV infection is categorized as a binary outcome, classifying the sex workers into either HIV–positive or HIV–negative groups. Similarly, hepatitis B infection is also a binary outcome, dividing the sex workers into those who are reactive and those who are non-reactive.

#### Independent variables.

Independent variables were selected based on a conceptual framework informed by prior literature on HBV and HIV risk among FSWs, emphasizing socio-demographic, behavioural, and healthcare access factors [7]. Socio-demographic variables included age (categorized as 16–24, 25–35, > 35 years), educational level (none, primary, middle/JSS/JHS, secondary, higher), and nationality (Ghana, Nigeria, Others). Age categories were chosen to reflect life stages associated with varying sexual risk behaviours and healthcare access, as younger FSWs (16–24) may face different vulnerabilities (e.g., less negotiation power) compared to older groups (25–35, > 35), who may have cumulative exposure risks [26,27]. Education and nationality were included due to their established associations with health literacy and migration-related risks, respectively [28,29]. Behavioural factors included condom use frequency (male condoms), marital status, weekly client volume, alcohol consumption during sex, and anal sex, as these are linked to HBV/HIV transmission through unprotected or high-risk sexual practices [30,31]. Healthcare access variables, such as mobility, sex work type (roamer versus seater), and avoidance of services due to stigma, were selected based on evidence of structural barriers impacting FSWs’ health-seeking behaviour [32].Other potential variables (e.g., income, drug use) were excluded because prior studies showed inconsistent or weaker associations with HBV/HIV in FSW populations, or data were unavailable in our survey [8,33]. Variable selection prioritized parsimony and relevance to the Ghanaian context, guided by stakeholder consultations and the National HIV and AIDS Strategic Plan [23].

### Model diagnostics

The Receiver Operating Characteristic (ROC) curve, along with the area under the curve (AUC), measures the model’s ability to discriminate between outcome categories. Higher AUC values indicate stronger discriminatory power [34]. Model fit can also be evaluated using information criteria such as the Akaike Information Criterion (AIC), where lower values indicate better trade-offs between fit and complexity [35].

#### Logistic regression model diagnostics.

Logistic regression diagnostics are essential for evaluating the model’s adequacy and predictive accuracy for binary outcomes. The Hosmer-Lemeshow test assesses goodness-of-fit by comparing observed and expected frequencies across deciles of predicted risk; a non-significant p-value suggests that the model fits the data well [34,35].

Pseudo-R² measures, such as McFadden’s R², offer a summary measure of the proportion of variance explained by the model, providing a rough analogue to R² in linear regression. Additional tools, including the likelihood ratio test, chi-squared test, can further support model evaluation [35,36].

#### Poison regression model diagnostics.

The modified Poisson regression model was evaluated using standard diagnostic tools, including deviance, Pearson chi-squared, and the Akaike Information Criterion (AIC) to assess model fit and predictive performance for binary outcomes such as disease prevalence. These measures help determine how well the model captures outcome variability, while robust standard errors enhance the reliability of inference.

Although conventional Poisson regression is designed for count data, its modified form with robust variance estimation is well-suited for estimating relative risks in binary outcome settings. This approach, introduced by Zou [37], has demonstrated strong performance. Extensions using generalised estimating equations allow for analysis of clustered or correlated binary data, maintaining validity in terms of bias, type I error, and confidence interval coverage when at least 50 clusters are used [38,39]. For model validation, goodness-of-fit tests such as the normalised residual sum of squares have been recently recommended, with Hagiwara and Matsuyama [40] identifying it as a particularly effective method.

#### Cox regression model diagnostics.

The Cox regression model was evaluated using standard diagnostics, including the log-likelihood, Wald, Score (log-rank), and Likelihood Ratio tests, to assess model fit and variable significance for binary outcomes such as HBV or HIV infection status among female sex workers. These diagnostics evaluate how well the model differentiates between infected and uninfected individuals.

Although the Cox model is traditionally used for time-to-event data, it has been adapted for use in cross-sectional studies by assuming a constant risk period. This adaptation allows estimation of prevalence proportion ratios (PPRs), as supported by Barros and Hirakata [41], and recommended by Lee and Chia [42].

### Model selection and comparison

#### Criteria for model selection.

Model selection is a critical aspect of statistical analysis, particularly when developing predictive models for health outcomes such as Hepatitis B Virus (HBV) and Human Immunodeficiency Virus (HIV) among female sex workers (FSWs) in Ghana. Two widely used criteria for model selection are the Akaike Information Criterion (AIC) and the Bayesian Information Criterion (BIC).

#### Akaike information criterion (AIC).

The AIC is a measure used to compare different statistical models, balancing model fit and complexity. It is calculated as:


AIC=2k−2ln(L)

where k is the number of parameters in the model and L is the likelihood of the model [17]. A lower AIC value indicates a better-fitting model. The AIC is particularly useful in situations where multiple models are being compared, as it penalizes models with more parameters, thus discouraging overfitting [17].

#### Bayesian information criterion (BIC).

Similar to AIC, the BIC is another criterion for model selection, but it places a heavier penalty on model complexity. It is calculated as:


BIC=ln(n)k−2ln(L)

where n is the sample size [43]. Like AIC, a lower BIC value indicates a better model fit. The BIC is particularly useful when the sample size is large, as it tends to favour simpler models compared to AIC [43].

#### Comparative analysis of models.

In the context of this study, a comparative analysis of models was carried out to determine the most appropriate predictive models for evaluating factors influencing Hepatitis B and HIV infections among female sex workers (FSWs). Logistic regression, modified Poisson regression, and Cox proportional hazards regression models were each assessed using the AIC and BIC to establish their relative performance.

The final model was selected based on its ability to balance goodness-of-fit with predictive accuracy while minimizing overfitting. This approach ensures that the chosen model can be generalized to other settings and is robust in predicting infection risk among this high-risk population. Through this comparative analysis, insights were gained into the strengths and limitations of each model, contributing to the development of a more accurate and reliable predictive framework for guiding interventions and policy decisions.

#### Predictive performance evaluation.

Evaluating the predictive performance of statistical models is crucial for ensuring that they accurately reflect the relationships within the data and provide reliable predictions. Several metrics are commonly used to assess model performance, particularly in the context of predicting Hepatitis B Virus and Human Immunodeficiency Virus infections among female sex workers in Ghana.

#### Area under the receiver operating characteristic curve (AUC-ROC).

The AUC-ROC is a performance measurement for classification models at various threshold settings. The AUC-ROC quantifies a model’s ability to differentiate between positive and negative cases. An AUC of 0.5 indicates no discrimination (i.e., the model performs no better than chance), while an AUC of 1.0 indicates perfect discrimination.

#### Sensitivity and specificity.

Sensitivity (true positive rate) measures the proportion of actual positives correctly identified by the model, while specificity (true negative rate) measures the proportion of actual negatives correctly identified. These metrics are crucial for evaluating the performance of binary classification models, such as logistic regression, in predicting health outcomes.

#### Positive and negative predictive values.

Positive predictive value (PPV) is the proportion of positive results in the model that are true positives, while negative predictive value (NPV) is the proportion of negative results that are true negatives. These metrics provide insight into the practical utility of the model in a clinical setting, as they indicate how likely it is that a positive or negative test result reflects the actual condition.

## Results

### Prevalence of HBV and exploratory analysis of socio-demographic factors

The prevalence of Hepatitis B (HBV) among female sex workers (FSWs) in Ghana was found to be 6.53% (95% CI: [6.08%, 7.01%]), with 330 out of 5,052 participants testing positive for HBV. This finding highlights the relatively low but significant burden of HBV within this high-risk population. Chi-square analyses revealed a statistically significant association between age group and HBV status (*χ²* = 12.214, *p* = 0.047). Participants aged 25–35 years exhibited the highest proportion of HBV-positive cases at 55.07%. In contrast, other socio-demographic factors such as educational level, nationality, region of residence, religious affiliation, and weekly income showed no statistically significant associations with HBV status (Table 1).

**Table 1:** Distribution of socio-demographic factors, individual factors, and healthcare access characteristics among female sex workers by hepatitis B virus (HBV) status in Ghana.

Variables	Negative	Positive	Total (%)	Chi^2^ (P-Value)
	n = 4,722 (93.47%)	n = 330 (6.53%)	N (5,052)	
**Socio-demographic factors**				
**Age Group**				**12.214(0.0474)**
16-24 years	2,054 (43.49)	121 (36.56)	2,175 (43.04)	
25-35 year	2,243 (47.51)	182 (55.07)	2,425 (48.00)	
>35 years	425 (9.00)	28 (8.37)	453 (8.96)	
**Educational Level**				**0.0733(0.8341)**
None	374 (7.92)	24 (7.42)	398 (7.89)	
Primary	795 (16.85)	59 (17.75)	854 (16.90)	
Middle/JSS/JHS	1,800 (38.13)	123 (37.34)	1,924 (38.08)	
Secondar00y	1,493 (31.63)	106 (31.97)	1,599 (31.65)	
Higher	259 (5.48)	18 (5.52)	277 (5.48)	
**Country/Nationality**				**4.955(0.101)**
Ghana	3,911 (82.83)	292 (88.62)	4,203 (83.20)	
Nigeria	688 (14.58)	32 (9.61)	720 (14.25)	
Others	123 (2.60)	6 (1.77)	129 (2.54)	
**Region**				**2.3665(0.2533)**
Ahafo	101 (2.14)	7 (2.11)	108 (2.14)	
Ashanti	900 (19.06)	75 (22.72)	975 (19.30)	
Bono	262 (5.54)	17 (5.26)	279 (5.52)	
Bono East	102 (2.16)	5 (1.63)	107 (2.13)	
Central	256 (5.43)	30 (9.23)	287 (5.68)	
Eastern	546 (11.57)	14 (4.32)	561 (11.10)	
Greater	1,324 (28.05)	112 (34.02)	1,437 (28.44)	
North East	19 (0.41)	3 (0.86)	22 (0.43)	
Northern	117 (2.47)	6 (1.69)	122 (2.42)	
Oti	112 (2.37)	12 (3.60)	124 (2.45)	
Savannah	25 (0.54)	1 (0.35)	27 (0.53)	
Upper East	106 (2.24)	9 (2.76)	115 (2.27)	
Upper West	48 (1.02)	4 (1.20)	52 (1.03)	
Volta	118 (2.50)	1 (0.23)	119 (2.35)	
Western	653 (13.82)	30 (9.17)	683 (13.52)	
Western North	32 (0.69)	3 (0.85)	35 (0.70)	
**Religion**				**0.793(0.4791)**
Christian	3,893 (82.44)	278 (84.23)	4,171 (82.56)	
Moslem	616 (13.04)	38 (11.66)	654 (12.95)	
Traditionalist	34 (0.72)	1 (0.44)	36 (0.70)	
No-religion	179 (3.80)	12 (3.67)	191 (3.79)	
**Earn per week**				**0.5488(0.536)**
GHC0-GHC1000	4,515 (95.63)	312 (94.65)	4,828 (95.56)	
>GHC1000	206 (4.37)	18 (5.35)	224 (4.44)	
**Individual risk factors**				
**Paying client use condom**				**1.0941(0.4054)**
Not Always	453 (9.59)	25 (7.52)	478 (9.45)	
Always	4,269 (90.41)	305 (92.48)	4,574 (90.55)	
**Ever Married/Cohabited**				**0.2441(0.6702)**
Yes	1,980 (41.93)	145 (43.84)	2,124 (42.05)	
No	2,742 (58.07)	185 (56.16)	2,928 (57.95)	
Client per week				**0.4836(0.5675)**
<10	3,516 (74.46)	253 (76.77)	3,769 (74.61)	
11–20	772 (16.35)	52 (15.81)	824 (16.32)	
>20	434 (9.18)	24 (7.42)	458 (9.07)	
**Alcohol during sex**				**5.7561(0.1385)**
No	1,904 (40.32)	107 (32.28)	2,011 (39.80)	
Yes	2,818 (59.68)	223 (67.72)	3,041 (60.20)	
Anal sex				**1.8628(0.3056)**
Yes	462 (9.79)	26 (7.76)	488 (9.66)	
No	4,260 (90.21)	304 (92.24)	4,564 (90.34)	
**Access to healthcare**				
**Sex Work Type**				**14.5343(0.0624)**
Roamer	3,965 (83.97)	282 (85.48)	4,247 (84.07)	
Seater	757 (16.03)	48 (14.52)	805 (15.93)	
**Avoid Health care due to Stigma**				**7.531(0.111)**
No	4,429 (93.80)	295 (89.49)	4,725 (93.52)	
Yes	293 (6.20)	35 (10.51)	327 (6.48)	

Individual behavioural factors were also analysed for their potential association with HBV status. The results indicated no significant relationships between HBV status and condom use frequency, marital history, weekly client volume, alcohol use during sex, or participation in anal sex. Healthcare-related factors such as access to healthcare and stigma-driven healthcare avoidance were also examined. Neither the type of sex work (*p* = 0.062) nor avoidance of healthcare due to stigma (*p* = 0.111) demonstrated significant associations with HBV status (Table 1).

### Prevalence of HIV and exploratory analysis of socio-demographic factors

The prevalence of HIV among female sex workers (FSWs) in this study was 4.53% (95% CI: [3.46%, 5.92%], with 246 cases testing positive out of the total sample of 5,426.

Chi-square analysis revealed significant associations between HIV status and certain socio-demographic factors, specifically age group (*χ²* = 28.1584, p = 0.0147) and educational level (*χ²* = 13.2083, p = 0.0468). Within the age groups, FSWs aged 25–35 years exhibited the highest prevalence of HIV (54.54%), followed by those over 35 years (16.27%). Similarly, educational attainment revealed disparities, with FSWs having no education (14.21%) or only primary education (29.95%) demonstrating higher HIV prevalence compared to those with secondary (17.99%) or higher education (3.76%). In contrast, no significant associations were found between HIV status and region of residence (p = 0.2276), religion (p = 0.4248), or weekly earnings (p = 0.6915). Among individual behavioural factors, only condom use frequency demonstrated a significant association with HIV status, with a notable p-value of less than 0.05 (Table 2).

**Table 2:** Distribution of socio-demographic factors, individual factors, and healthcare access characteristics among female sex workers by HIV status in Ghana.

Variables	Negative	Positive	Total n (%)	Chi^2^ (P-value)
	n = 5,180 (95.47)	n = 246 (4.53)	N (5,426)	
**Socio-demographic factors**				
**Age Group**				**28.1584(0.0147)**
16-24 years	2,251 (43.45%)	72 (29.18%)	2,323 (42.81%)	
25-35 year	2,494 (48.15%)	134 (54.54%)	2,628 (48.44%)	
>35 years	435 (8.40%)	40 (16.27%)	475 (8.76%)	
**Educational Level**				**13.6803(0.0468)**
None	380 (7.34%)	35 (14.21%)	415 (7.65%)	
Primary	816 (15.75%)	74 (29.95%)	890 (16.40%)	
Middle/JSS/JHS	2,042 (39.43%)	84 (34.09%)	2,126 (39.19%)	
Secondary	1,666 (32.16%)	44 (17.99%)	1,710 (31.52%)	
Higher	276 (5.32%)	9 (3.76%)	285 (5.25%)	
**Country/Nationality**				**2.1262(0.2803)**
Ghana	4,357 (84.11%)	206 (83.86%)	4,563 (84.10%)	
Nigeria	700 (13.52%)	31 (12.73%)	732 (13.49%)	
Others	123 (2.37%)	8 (3.41%)	131 (2.42%)	
**Region**				**2.7202(0.2276)**
Ahafo	106 (2.04%)	3 (1.26%)	109 (2.01%)	
Ashanti	946 (18.26%)	39 (15.75%)	984 (18.14%)	
Bono	324 (6.25%)	13 (5.19%)	337 (6.21%)	
Bono East	100 (1.94%)	8 (3.26%)	108 (2.00%)	
Central	546 (10.54%)	14 (5.66%)	560 (10.32%)	
Eastern	557 (10.76%)	23 (9.46%)	581 (10.70%)	
Greater	1,350 (26.05%)	77 (31.24%)	1,426 (26.29%)	
North East	22 (0.43%)	0 (0.00%)	22 (0.41%)	
Northern	120 (2.32%)	3 (1.34%)	123 (2.27%)	
Oti	125 (2.41%)	1 (0.49%)	126 (2.32%)	
Savannah	26 (0.49%)	1 (0.49%)	27 (0.49%)	
Upper East	106 (2.04%)	10 (4.06%)	116 (2.13%)	
Upper West	48 (0.92%)	5 (2.02%)	53 (0.97%)	
Volta	114 (2.19%)	6 (2.54%)	120 (2.21%)	
Western	658 (12.70%)	40 (16.33%)	698 (12.86%)	
Western North	33 (0.64%)	2 (0.91%)	36 (0.66%)	
**Religion**				**0.998(0.4248)**
Christian	4,310 (83.21%)	190 (77.23%)	4,500 (82.94%)	
Moslem	640 (12.35%)	42 (17.12%)	682 (12.57%)	
Traditionalist	34 (0.65%)	2 (0.86%)	36 (0.66%)	
No-religion	197 (3.79%)	12 (4.78%)	208 (3.84%)	
**Earn per week**				**0.2534(0.6915)**
GHC0-GHC1000	4,958 (95.72%)	234 (95.26%)	5,193 (95.70%)	
>GHC1000	222 (4.28%)	12 (4.74%)	233 (4.3%)	
**Individual risk factors**				
**Paying client use condom**				**0.2196(0.6855)**
Not Always	519 (10.02%)	22 (8.79%)	541 (9.97%)	
Always	4,661 (89.98%)	224 (91.21%)	4,885 (90.03%)	
**Ever Married/Cohabited**				**7.1789(0.1156)**
Yes	2,204 (42.54%)	120 (48.59%)	2,323 (42.81%)	
No	2,976 (57.46%)	126 (51.41%)	3,103 (57.19%)	
**Client per week**				**13.7853(0.0586)**
<10	3,901 (75.30%)	183 (74.37%)	4,084 (75.26%)	
11–20	837 (16.15%)	30 (12.02%)	866 (15.96%)	
>20	443 (8.54%)	34 (13.61%)	476 (8.77%)	
**Alcohol during sex**				**0.064(0.824)**
No	2,046 (39.49%)	95 (38.50%)	2,140 (39.45%)	
Yes	3,134 (60.51%)	151 (61.50%)	3,286 (60.55%)	
**Anal sex**				**0.0583(0.8317)**
Yes	523 (10.10%)	23 (9.16%)	546 (10.05%)	
No	4,657 (89.90%)	223 (90.84%)	4,880 (89.95%)	
**Access to healthcare**				
**Sex Work Type**				**0.1266(0.756)**
Roamer	4,385 (84.65%)	210 (85.40%)	4,595 (84.69%)	
Seater	795 (15.35%)	36 (14.60%)	831 (15.31%)	
**Avoid Health care due to Stigma**				**0.5788(0.5263)**
No	4,850 (93.63%)	232 (94.33%)	5,082 (93.66%)	
Yes	330 (6.37%)	14 (5.67%)	344 (6.34%)	
**Comprehensive Knowledge of HIV and AIDS**				**1.3254(0.3687)**
Yes	1,763 (34.03%)	79 (31.98%)	1,841 (33.93%)	
No	3,417 (65.97%)	167 (68.02%)	3,585 (66.07%)	

## Model evaluation

### HBV

The Cox regression model provided the best fit for assessing HBV risk among female sex workers, outperforming both logistic and modified Poisson regression models across multiple metrics. The Cox model had the lowest Akicaike Information Criterion (AIC = 5923.345) and Bayesian Information Criterion (BIC = 5831.959), indicating better model parsimony and predictive ability compared to alternatives (Table 3). The model’s C-index was 0.599, suggesting moderate ability to correctly rank individuals by HBV risk.

**Table 3:** Comparison of model performance metrics for HBV and HIV using logistic regression, modified poisson, and cox regression models using AIC, BIC, C-Index, ROC, sensitivity, and specificity.

HBV						
**Model**	**AIC**	**BIC**	**C-Index (Cox)**	**AUC-ROC**	**Sensitivity**	**Specificity**
Logistic Regression	22123.4	22221.31	NA	0.590	0.967	0.057
Modified Poisson	22347.08	22444.99	NA	0.590	0.977	0.033
Cox Regression	5923.345	5831.959	0.599	0.590	0.972	0.085
Threshold					0.1	
**HIV**						
**Model**	**AIC**	**BIC**	**C-Index (Cox)**	**AUC-ROC**	**Sensitivity**	**Specificity**
Logistic Regression	17479.74	17572.12	NA	0.6483	0.955	0.130
Modified Poisson	17624.35	17716.74	NA	0.6484	0.959	0.123
Cox Regression	2323.845	2409.631	0.6699	0.6476	0.970	0.741
Threshold					0.1	

For classification, the Cox model showed a high sensitivity of 0.972, meaning it correctly identified the majority of FSWs who tested positive for HBV. However, specificity was much lower (0.085), indicating limited accuracy in identifying true negatives. This imbalance highlights a limitation: while the model is efficient at flagging at-risk individuals, it also produces a high number of false positives, which could lead to over-targeting and misallocation of public health resources. This trade-off is further reflected in the Area Under the Receiver Operating Characteristic curve (AUC-ROC), which was 0.590, identical to logistic and Poisson models, indicating moderate discrimination overall.

The evaluation applied a risk threshold of 0.1 across all models. This decision prioritizes sensitivity, aligning with public health goals to minimize missed infections in high-risk populations. However, it comes at the cost of specificity, reinforcing the need for careful program planning when translating predictions into interventions (Table 3).

### HIV

The Cox regression model also outperformed logistic and modified Poisson regression models in predicting HIV risk. It achieved the lowest AIC (2323.845) and BIC (2409.631), supporting its superior fit (Table 3). The model demonstrated a C-index of 0.670, indicating a good ability to rank individuals by HIV risk. Its AUC-ROC was 0.648, which is slightly higher than those of the logistic (0.6483) and Poisson (0.6484) models and shows acceptable discriminatory power.

Unlike in the HBV results, the Cox model demonstrated a better balance between sensitivity and specificity for HIV. It achieved a high sensitivity of 0.970, capturing nearly all true positives, and a substantially higher specificity of 0.741 compared to other models. This indicates the Cox model not only identifies individuals at risk but also reasonably distinguishes those unlikely to be infected. In contrast, the logistic and Poisson models exhibited lower specificity (0.130 and 0.123), suggesting a higher false positive rate and thus limited utility in resource-targeted settings.

The same 0.1 threshold was applied to the HIV models. In this case, the Cox model performs well in both directions—detecting infections while limiting misclassification. This makes it more suitable for operational use in identifying high-priority FSWs for intervention programs (Table 3).

## Cox proportional hazards analysis

### HBV

The Cox proportional hazards model revealed significant risk factors for HBV among female sex workers (FSWs). Nationality was an important determinant, with Nigerian FSWs having a 38% lower risk of HBV compared to Ghanaian FSWs (aHR: 0.62, 95% CI [0.40, 0.95], *p* = 0.027). Alcohol use was associated with increased risk, as FSWs who consumed alcohol during sexual encounters experienced a 34% higher risk of HBV compared to non-drinkers (aHR: 1.34, 95% CI [1.01, 1.77], *p* = 0.042). Similarly, FSWs who avoided healthcare services due to stigma had a 64% higher risk of HBV (aHR: 1.64, 95% CI [1.10, 2.42], *p* = 0.023). These findings highlight the significant influence of structural and behavioural factors on HBV risk in this population, particularly stigma and alcohol consumption. Other factors, including age, education, condom use, number of clients per week, and time spent in sex work, were not significantly associated with HBV risk in this model (Table 4).

**Table 4:** Cox proportional hazard regression analysis of factors associated with HBV among female sex workers.

Variables	HBV aHR [95% CI]	p-value
**Age Group**		
16-24 years	ref	
25-35 years	1.33 [0.98, 1.79]	0.064
>35 years	1.15 [0.72, 1.84]	0.556
**Education**		
None (ref)	ref	
Primary	1.15 [0.69, 1.94]	0.583
Middle/JSS/JHS	1.05 [0.66, 1.67]	0.822
Secondary/SSS/SHS	1.19 [0.75, 1.93]	0.463
Higher	1.16 [0.61, 2.19]	0.671
**Country/ Nationality**		
Ghana (ref)	ref	
Nigeria	0.62 [0.40, 0.95]**	0.027
Others	0.65 [0.23, 1.84]	0.443
**Condom Use**		
No (ref)	ref	
Yes	1.37 [0.90, 2.10]	0.163
**Clients per Week**		
<10 (ref)	ref	
11–20	0.90 [0.62, 1.31]	0.566
>20	0.84 [0.52, 1.36]	0.5
**Alcohol Use**		
No (ref)	ref	
Yes	1.34 [1.01, 1.77]**	0.042
**Time Spent on Sex Work**		
Roamer (ref)	ref	
Seater	0.90 [0.63, 1.30]	0.586
**Avoidance of Services**		
No (ref)	ref	
Yes	1.64 [1.10, 2.42]**	0.023

### HIV

In the Cox regression analysis of HIV status, several variables demonstrated significant associations with the hazard of HIV incidence. Age was a strong predictor, with individuals aged 25–35 years having a 1.60 times higher risk of developing HIV compared to those aged 16–24 years (aHR = 1.60, 95% CI [1.27, 2.02]; p = 0.007). Those older than 35 years had an even greater risk, with a hazard ratio of 2.20 (aHR = 2.20, 95% CI [1.55, 3.14]; p = 0.001), suggesting that both advancing in age had a higher likelihood of HIV onset among older female sex workers. Education also emerged as a significant factor. Individuals with middle, junior secondary, or high school education had a 49% reduced risk of HIV (aHR = 0.51, 95% CI [0.35, 0.73]; p = 0.003), while those with secondary education (aHR = 0.33, 95% CI [0.19, 0.53]; p < 0.001) and higher education (aHR = 0.40, 95% CI [0.19, 0.85]; p = 0.019) had even lower risks. These results indicate that higher levels of education are protective against HIV infection among this population (Table 5).

**Table 5:** Cox proportional hazard regression analysis of factors associated with HBV among female sex workers.

Variables	HIV aHR [95% CI]	p-value
**Age Category**		
16-24 years	ref	–
25-35 years	1.60 [1.27, 2.02]	0.007*
>35 years	2.20 [1.55, 3.14]	0.001*
**Education**		
None	ref	–
Primary	1.07 [0.66, 1.70]	0.79
Middle/JSS/JHS	0.51 [0.35, 0.73]	0.003*
Secondary/SSS/SHS	0.33 [0.19, 0.53]	<0.001*
Higher	0.40 [0.19, 0.85]	0.019*
**Condom Use**		
No	ref	–
Yes	1.16 [0.68, 1.97]	0.593
**Ever Married**		
Yes	ref	–
No	0.99 [0.73, 1.34]	0.937
**Client Week**		
<10	ref	–
11–20	0.74 [0.47, 1.16]	0.167
>20	1.61 [0.98, 2.66]	0.065
**Alcohol Use**		
No	ref	–
Yes	1.06 [0.78, 1.41]	0.712
**Avoid Service Stigma**		
No	ref	–
Yes	0.94 [0.53, 1.68]	0.844
**Comprehensive Knowledge**		
Yes	ref	–
Not Yes	0.98 [0.73, 1.34]	0.923

## Discussion

### HBV

The prevalence of Hepatitis B Virus (HBV) among female sex workers (FSWs) in Ghana is a crucial indicator of the burden of this viral infection in a high-risk population. The 6.53% prevalence rate (95% CI: [6.08%, 7.01%]) provides important insight into the scope of HBV among FSWs in the country. This relatively low prevalence might be attributed to the potential effectiveness of interventions such as vaccination programs and the limited sample size compared to the broader population. However, the prevalence still remains a public health concern, given the vulnerabilities of FSWs to HBV transmission due to factors like multiple sexual partners, inconsistent condom use, and limited access to healthcare.

In examining the socio-demographic factors, the exploratory analyses revealed a significant association between age and HBV status, with the highest proportion of HBV-positive cases found in the 25–35 years age group (55.07%). This finding aligns with other studies that have suggested that younger to middle-aged populations are at greater risk of HBV infection, possibly due to higher engagement in risky sexual practices [44]. It is also consistent with the understanding that HBV prevalence can peak in sexually active and reproductive years, as these individuals are more likely to engage in multiple sexual encounters, thereby increasing their risk of exposure [45,46]. However, no significant associations were found between other socio-demographic factors such as educational level, nationality, region of residence, religion, and weekly income with HBV status. This lack of association may indicate that despite differences in socio-economic factors, these do not necessarily correlate with the likelihood of HBV infection in this specific population.

Behavioral factors, such as condom use frequency, marital history, and alcohol use during sex, were also analyzed for their potential influence on HBV risk. While a higher frequency of condom use was observed among those who tested negative for HBV, this factor did not reach statistical significance in the chi-squared test. These results suggest that despite a high awareness of safe sex practices among FSWs, condom use may not be consistently adhered to, which has been linked to a higher risk of HBV and other sexually transmitted infections (STIs) [31]. Similarly, the lack of significant findings for alcohol use during sex or participation in anal sex in relation to HBV status may reflect a complex interplay of behavioral, structural, and biological factors that are not easily captured by simple categorical variables. However, the Cox regression model did find alcohol use during sex to be significantly associated with a 34% increased risk of HBV infection, highlighting the role of substance use in risky sexual behaviors [30,47–50].

The model evaluation revealed that the Cox proportional hazards model provided the best fit for assessing HBV risk. This model’s lower Akaike Information Criterion (AIC) and Bayesian Information Criterion (BIC) values, compared to logistic regression and modified Poisson models, suggest superior parsimony and predictive accuracy [51–53]. The Cox model also demonstrated a high sensitivity (97.3%), indicating its effectiveness in identifying individuals at risk of HBV, which is crucial for public health initiatives aimed at preventing transmission within high-risk populations. However, the low specificity (8.5%) suggests that the model may not be as effective at identifying those who are not at risk, which can be a limitation when designing targeted interventions.

The significant findings from the Cox proportional hazards analysis provide valuable insights into the risk factors for HBV among FSWs. The lower risk of HBV among Nigerian FSWs compared to Ghanaian FSWs (aHR: 0.62, 95% CI [0.40, 0.95], p = 0.027) may reflect regional differences in healthcare access, cultural practices, or sexual health interventions [28]. Additionally, the increased risk associated with alcohol use during sex (aHR: 1.34, 95% CI [1.01, 1.77], p = 0.042) and the avoidance of healthcare due to stigma (aHR: 1.64, 95% CI [1.10, 2.42], p = 0.023) are critical findings. These factors highlight the importance of addressing both individual and structural barriers to healthcare access. Stigma-driven avoidance of healthcare has been widely documented as a key factor hindering FSWs’ ability to access HIV and HBV prevention and treatment services [32].

In conclusion, the findings from this study emphasize the importance of considering both behavioral and structural determinants of HBV risk in FSW populations. The significant association between age and HBV status, along with the identified risks related to alcohol use and healthcare avoidance, point to areas where targeted interventions could be most effective. Further research is needed to explore these relationships in greater depth, particularly focusing on strategies to mitigate the stigma surrounding healthcare services and the role of alcohol in increasing HBV risk. Public health efforts should also continue to prioritize vaccination programs and condom distribution to reduce HBV transmission in high-risk groups like FSWs.

### HIV

The prevalence of HIV among female sex workers (FSWs) in this study was found to be 4.53%, which is consistent with findings from previous studies reporting similar rates within the FSW population in sub-Saharan Africa [54]. The exploratory analyses of socio-demographic factors identified significant associations between HIV status and age as well as educational level, reflecting the complex interplay of socio-economic and behavioral factors in HIV risk [55–57].

Age emerged as a critical factor influencing HIV prevalence, with the highest rates observed among FSWs aged 25–35 years (54.54%), followed by those over 35 years (16.27%). This result aligns with findings that observed higher HIV risks with advancing age among FSWs [26,27,58]. The increase in HIV prevalence with age can be attributed to longer exposure to high-risk sexual behaviors, reduced condom use over time, and potential co-morbidities associated with aging, as described by [59,22]. Moreover, the Cox regression model demonstrated that individuals aged 35 and older had a significantly higher risk of contracting HIV, suggesting a cumulative effect of age on HIV vulnerability, consistent with findings from [55].

Education also played a significant role in determining HIV risk, with higher education being protective against HIV infection. FSWs with no formal education or only primary education exhibited higher HIV prevalence (14.21% and 29.95%, respectively) compared to those with secondary or higher education. This is consistent with several studies that highlight the protective effect of education on sexual health outcomes [29,60,61]. Higher education often correlates with better access to health information, increased awareness of HIV prevention methods, and greater autonomy in negotiating safer sexual practices. This suggests that education is a key factor in reducing HIV risk among FSWs [27].

Despite these significant findings, region of residence, religion, and weekly earnings did not show any significant associations with HIV status. This contrasts with other studies that have suggested that regional differences may influence HIV transmission dynamics due to varying levels of healthcare access and social stigma [62]. Similarly, studies found no strong relationship between religion and HIV status among high-risk populations, suggesting that socio-cultural factors may play a less direct role in HIV acquisition than other behavioral and demographic factors [33].

One individual risk factor that showed a significant association with HIV status was condom use. FSWs who consistently used condoms had a significantly lower risk of HIV infection. This is in line with multiple studies that emphasize the importance of consistent condom use in HIV prevention, particularly among FSWs [63]. However, the high prevalence of condom use among FSWs in this study (approximately 90%) contrasts with findings from other regions where condom use is less frequent, suggesting regional differences in access to condoms and sexual health education [64–66].

The results from the Cox regression analysis further support the importance of both age and education in predicting HIV risk. Older age and lower education levels were significantly associated with a higher hazard of HIV acquisition. Specifically, FSWs aged 25–35 years had a 1.60 times higher risk of HIV, and those older than 35 years had a 2.20 times higher risk. These results align with findings by [26], who observed a similar trend in age-related HIV vulnerability.

Interestingly, no significant associations were found between HIV status and variables such as alcohol use, ever being married, or the number of clients seen per week. This suggests that these factors, while important in other studies, may not be as strongly correlated with HIV risk in this specific population. For instance, alcohol use may increase HIV risk by impairing judgment and reducing condom use, but the absence of a significant finding in this study may reflect a lack of robust reporting or differences in behavioural context, as suggested by [67].

Finally, the Cox regression model outperformed both logistic regression and modified Poisson models in predicting HIV risk among FSWs. The Cox model achieved a higher C-Index of 0.6699, indicating better ranking of individuals by their HIV risk. This is consistent with findings that highlighted the superior predictive capabilities of Cox models in epidemiological studies of risk by [68]. The higher sensitivity (0.9702) and specificity (0.7415) of the Cox model underscore its ability to correctly identify both high- and low-risk individuals, thus offering an effective tool for targeted interventions.

### Recommendations for policy based on HBV and HIV findings

- **Health Education and Awareness:** Implement targeted educational campaigns addressing alcohol use, safe sexual practices, and age-specific interventions, with a focus on HBV vaccination and regular screenings for both HBV and HIV, particularly for FSWs aged 25–35 years.
- **Improve Healthcare Access:** Reduce stigma in healthcare settings through provider training and establish dedicated clinics for FSWs to ensure access to services without judgment, focusing on privacy and rights protection.
- **HBV Vaccination and Screening:** Launch targeted HBV vaccination programs for high-risk FSWs, especially those aged 25–35, and advocate for regular HBV screening and booster shots to identify cases early and reduce transmission.
- **Promote Safer Sex and Risk Reduction:** Strengthen condom availability and usage through community programs, engage male clients in safe sex education, and incorporate alcohol reduction programs and behavioural counselling to reduce HBV and HIV transmission risks.

### Study limitations

This study has several limitations that should be considered when interpreting its findings. First, the use of Time Location Sampling (TLS) ensured a representative sample across Ghana’s 16 regions; however, it may have missed FSWs who do not frequent identified venues or operate during non-peak times, potentially introducing selection bias. Additionally, the reduction in sample size for HBV (N = 5,052) and HIV (N = 5,426) analyses from the 7,000 recruited FSWs, due to missing data in behavioural and biological variables, may limit the generalizability of findings and potentially bias the results towards participants with complete data (see Sample Size and Data Completeness for details). The reliance on self-reported behavioural data, such as male condom use, alcohol consumption during sex, and healthcare avoidance due to stigma, may be subject to recall bias or social desirability bias, which could affect the accuracy of reported risk behaviours.

Additionally, the focus on FSWs aged 16 years and older, though aligned with the Ghana AIDS Commission’s definition, may exclude younger adolescents engaged in sex work, limiting the study’s applicability to this subgroup. Finally, the exclusion of Hepatitis C Virus (HCV) testing due to resource constraints, as noted in the background, restricts the study’s ability to provide a comprehensive picture of sexually transmitted infections among FSWs.

### Conclusions

The findings from this study highlight the significant public health concerns posed by both Hepatitis B Virus (HBV) and HIV among female sex workers (FSWs) in Ghana, with nuanced differences in risk factors and model performance. For HBV, the study found a relatively low prevalence of 6.53%, but the burden remains substantial within this high-risk population. Cox regression analyses identified several key factors associated with HBV infection. Notably, age (particularly the 25–35 years group) and alcohol use during sex emerged as significant risk factors, suggesting that behavioral factors such as substance use may amplify the risk of viral transmission.

For HIV, while similar demographic and behavioral factors influence risk, the findings were generally in line with existing literature on HIV in FSW populations. Similar behavioral dynamics—such as inconsistent condom use, alcohol consumption, and healthcare access issues—likely play a critical role in HIV transmission risk among FSWs in Ghana.

The model comparison revealed that the Cox regression model was the best-performing approach for assessing HBV risk, demonstrating high sensitivity but lower specificity. This suggests the model’s strength lies in identifying those at risk, which is crucial for targeted intervention programs. The comparative analysis of HBV and HIV using different statistical models, including logistic regression, modified Poisson, and Cox regression, points to the importance of using robust statistical approaches to accurately assess the risk profiles in vulnerable populations.

Further research should aim to explore the complex interplay of structural, behavioral, and socio-demographic factors in shaping HBV and HIV risks, and to refine predictive models that can better identify individuals at risk.

## Supporting information

S1 File Statistical considerations. (PDF)
