# Supplementary material for: Evaluation of statistical regression models in predicting factors influencing HBV and HIV among female sex workers in Ghana: A Bio-behavioural survey
Source: PLoS One. 2025 Sep 12;20(9):e0332152. doi: 10.1371/journal.pone.0332152 (PMC12431487; doi:10.1371/journal.pone.0332152)
Supplement: S1 File — (PDF) [file pone.0332152.s001.pdf]

STATISTICAL CONSIDERATIONS / METHODOLOGY

Introduction

This supplementary outline the statistical considerations and methodologies utilized in evaluating parametric statistical methods for predicting factors influencing Hepatitis B (HBV) and HIV among female sex workers (FSWs). It begins by detailing the rationale for selecting specific statistical techniques, such as logistic regression, Poisson regression, and Cox regression models.

Theoretical Foundation of Parametric Methods

Logistic Regression

Theoretical Background

Logistic regression is employed when the dependent variable is categorical, particularly in cases where it is binary, such as the presence or absence of HBV or HIV infection. Binary logistic regression specifically addresses scenarios where the outcome variable has two possible outcomes. However, logistic regression can also extend to multinomial logistic regression, which is applicable when the dependent variable has more than two categories. This method models the probability of the outcome occurring as a function of one or more predictor variables, using the logistic function to ensure that predicted probabilities fall between 0 and 1 (Hosmer & Lemeshow, 2000).

The logistic regression model is formulated as follows:

$$P(Y=1|X) = \frac{1}{1+e^{-(\beta_0+\beta_1 X_1+\cdots+\beta_k X_k)}}$$

Here, $P(Y=1|X)$ represents the probability of infection given the predictor variables$X_1, X_2, \ldots, X_k$. The logit transformation allows for this relationship to be expressed in a linear form:

$$\log\left(\frac{P(Y=1|X)}{1-P(Y=1|X)}\right)=\beta_0+\beta_1X_1+\cdots+\beta_kX_k$$

where β_0 is the intercept and $\beta_1, \beta_2, \ldots, \beta_k$ are the coefficients of the explanatory variables.

Assumptions

Logistic regression requires several assumptions:

Independence of Observations: Each observation should be independent of the others, a requirement that is often challenged in studies involving social networks, where behaviours may be influenced by connected individuals (Matthews *et al.*, 2024).

Linearity in the Logit: The relationship between the independent variables and the log odds of the dependent variable should be linear. This can be assessed using the Box-Tidwell test or by examining interaction terms (Box & Tidwell, 1962).

Absence of Multicollinearity: Absence of multicollinearity is essential, as independent variables should not be highly correlated with each other. Multicollinearity, which can distort regression analysis, can be assessed using several methods, including Variance Inflation Factors (VIF), with values above 10 indicating potential issues (Upendra *et al.*, 2023).

Model Specification: Model specification in logistic regression involves selecting appropriate independent variables that influence the likelihood of infection. For example, factors such as age, education, and risk behaviours should be included based on prior research.

Parameter Estimation

Parameter estimation in logistic regression is typically performed using maximum likelihood estimation (MLE), which finds the parameter values that maximize the likelihood of observing the given data (Hosmer & Lemeshow, 2000).

The likelihood function for the observed data is given by:

$$L(\beta) = \prod_{i=1}^{n} P(Y_i|X_i)^{Y_i}[1 - P(Y_i|X_i)]^{1-Y_i}$$

Maximizing the log-likelihood function provides the estimates for the coefficients $\beta_0, \beta_1, \beta_2, \ldots, \beta_k$ in logistic regression. These coefficients represent the change in the log odds of the outcome for a one-unit increase in the corresponding continuous explanatory variable. For categorical variables, the coefficients indicate the change in log odds relative to a reference category, providing a comparative assessment of the impact of different categories on the outcome.

Interpretation of Coefficients

The coefficients in logistic regression have a meaningful interpretation in terms of odds ratios:

The intercept β_0 represents the log odds of the outcome when all explanatory variables are zero.

Each coefficient β_j represents the change in the log odds of the outcome for a one-unit increase in the explanatory variable X_j when X_j is a continuous variable. For categorical variables, the interpretation of the coefficients is relative to a reference category, meaning that each coefficient indicates the change in log odds for being in a particular category compared to the reference category. The odds ratio for X_j is given by e^{β_j}, which indicates how the odds of the outcome change with a one-unit increase in X_j for continuous variables, or how the odds change when moving from the reference category to the category represented by X_j for categorical variables.

Poisson Regression

Theoretical Background

Poisson regression is a generalized linear model (GLM) designed for modelling count data, where the outcome represents the number of events that occur within a fixed time period or space. It is particularly useful in predicting the incidence of infections like Hepatitis B (HBV) and HIV among Female Sex Workers (FSWs) by modelling the rate of infections over a defined period. In this context, Poisson regression accounts for the count nature of the outcome and allows for a log-linear relationship between the incidence rate and the predictor variables.

While Poisson regression is primarily used for count outcomes, it can also be adapted for binary outcomes using quasi-likelihood models, especially in cases of overdispersion. Robust standard errors can enhance the reliability of the model's inference (McCullagh & Nelder, 1989; Green, 1984; Agresti, 2015; McCullagh, 1983; Wedderburn, 1974).

Assumptions

Poisson regression is based on several key assumptions:

Independence of Observations: Similar to other regression methods, observations should be independent. This is crucial to avoid bias in estimating the incidence rates

Equality of Mean and Variance: In Poisson regression, the mean of the response variable is assumed to be equal to its variance. If this assumption is violated, alternative methods, such as negative binomial regression, may be more appropriate.

Model Specification

Model specification involves selecting the appropriate independent variables that influence the count of infections. Relevant predictors may include risk behaviours, socio-economic factors, and access to healthcare services.

Parameter Estimation

Parameter estimation in Poisson regression is typically conducted using MLE, similar to logistic regression. The estimated coefficients represent the expected change in the log count of the outcome variable for a one-unit change in the predictor variable.

The Poisson regression model assumes that the expected count λ is related to the predictor variables via a log-link function:

$$log(\lambda_I) = \beta_0 + \beta_1 X_{i1} + \cdots + \beta_k X_{ik}$$

where λ_i represents the expected count for individual i, and $X_{i1}, X_{i2}, \ldots, X_{ik}$, are the explanatory variables. Maximum Likelihood Estimation (MLE) is used to estimate the regression coefficients (β_0, β_1,β_2,…,β_k) by maximizing the likelihood function:

$$L(\beta) = \prod_{i=1}^{n} \frac{e^{-\lambda_i} \lambda_i^{y_i}}{y_i!}$$

The log-likelihood function simplifies the estimation process:

$$logL(\beta) = \sum_{i=1}^{n} [y_i(\beta_0 + \beta_1 X_{i1} + \cdots + \beta_k X_{ik}) - e^{\beta_0 + \beta_1 X_{i1} + \cdots + \beta_k X_{ik}} - \log(y_i!)]$$

The log-likelihood is maximized with respect to the coefficients to obtain their estimates, allowing inference about the relationship between the explanatory variables and the infection rates. The estimated coefficients represent the expected change in the log count of the outcome variable for a one-unit change in the predictor variable.

Quasi-Likelihood Estimation

When the strict assumptions of Poisson regression (such as equidispersion) are violated, quasi-likelihood estimation provides a flexible alternative. Quasi-likelihood does not assume that the

data follows a fully specified probability distribution; rather, it focuses on the relationship between the mean μ and the variance $V(\mu)$. This method is particularly useful in the presence of overdispersion, where the variance exceeds the mean.

The quasi-likelihood function is given by:

$$Q(y,\mu) = \int \frac{y-\mu}{V(\mu)} \partial\mu$$

where $\boldsymbol{y}$ is the observed outcome, μ is the expected mean, and $V(\mu)$ represents the variance as a function of the mean. In quasi-likelihood estimation, the quasi-score function is used to estimate the parameters by solving:

$$\sum_{i=1}^{n} \frac{y_i - u_i}{V(\mu_i)} . \frac{\partial\mu_i}{\partial\beta} = 0$$

This approach allows for robust estimation of parameters even when the exact distributional assumptions of the Poisson model are not satisfied. This flexibility makes quasi-likelihood particularly suitable for modelling count data with overdispersion in studies of Hepatitis B and HIV among FSWs, where the data may not adhere strictly to Poisson assumptions.

Robust Standard Errors

When model assumptions like equidispersion are violated, robust standard errors provide more reliable inferences. The sandwich estimator adjusts the standard errors to account for misspecification or overdispersion:

$$\hat{V}_R = (X^T W X)^{-1} X^T W \hat{D} W X (X^T W X)^{-1}$$

where **X** is the matrix of explanatory variables, W is a diagonal matrix of weights, and $\hat{D}$ contains the squared residuals divided by the predicted values. This method improves the estimation of

standard errors and ensures valid inference even when the variance structure deviates from the Poisson assumptions (Cameron & Trivedi, 2013).

Interpretation of Coefficients

The coefficients in Poisson regression are interpreted as follows:

The intercept β_0 represents the log of the expected count when all predictors are at their reference levels (typically zero).

Each coefficient β_j the change in the log of the expected count for a one-unit increase in the corresponding predictor variable X_j . The exponentiated coefficients e^{β_j} indicate the multiplicative change in the expected count for a one-unit change in X_j.

Cox regression model

Theoretical Background

The Cox proportional hazards model is traditionally a semiparametric model used for survival analysis, examining the relationship between time to an event (e.g., infection, death) and explanatory variables. In this study on predicting Hepatitis B and HIV among female sex workers (FSWs), this model can be adapted for binary outcomes by treating infection status (e.g., infected or not infected) as the event of interest. Here, the time component becomes constant, and we focus on the risk factors influencing the likelihood of infection.

Model Formulation

The Cox proportional hazards model evaluates the relationship between the event occurrence (in this case, infection by Hepatitis B or HIV) and the covariates. The hazard function $h(t \mid X)$ describes the instantaneous risk of infection occurring at time t, given a set of covariates X.

For binary outcomes, the event either occurs (infected) or does not occur (not infected). The model can still be formulated as:

$$h(t \mid X) = h_0(t) exp(\beta_1 X_1 + \beta_2 X_2 + \cdots + \beta_k X_k)$$

Where:

- $h(t \mid X)$ is the hazard function, or the risk of infection at time t given the covariates X.
- $h_0(t)$ is the baseline hazard function.
- $X_1, X_2, \cdots, X_k$, are the covariates influencing the risk of infection (e.g., age, condom use, number of sexual partners).
- $\beta_1, \beta_2, \cdots, \beta_k$ are the coefficients representing the effect of each covariate on the hazard rate.

Even with fixed covariates (as in binary outcomes), the model's framework remains useful for evaluating the relative hazard, i.e., the risk of infection across different levels of predictors.

Hazard Ratio

The hazard ratio (HR) in the Cox model compares the relative risk of the event (infection) between two individuals or groups with different covariates. If we have two different sets of covariates X and X', the hazard ratio is:

$$\frac{h(t|X)}{h(t|X')} = \exp\left(\beta_1(X_1 - X_1') + \beta_2(X_2 - X_2') + \cdots + \beta_k(X_k - X_k')\right)$$

- A hazard ratio greater than 1 suggests an increased risk of infection for the individual with covariates X.
- A hazard ratio less than 1 suggests a decreased risk of infection.
- A hazard ratio equal to 1 indicates no difference in risk.

Partial Likelihood Estimation

The parameters β in the Cox model are estimated using partial likelihood, which does not require specifying the baseline hazard function $h_0(t)$. This makes the Cox model semiparametric. The partial likelihood function for n individuals is:

$$L(\beta) = \prod_{i=1}^{n} \frac{\exp{(\beta^T X_i)}}{\sum_{j \epsilon R(t_i)} \exp{(\beta^T X_j)}}$$

where:

- $R(t_i)$ is the risk set, consisting of all individuals who are at risk just before time t_i
- The numerator is the exponential function of the covariates for the individual iii, while the denominator sums the exponential functions of the covariates for all individuals at risk at time t_i

The log-partial likelihood is then:

$$\log L(\beta) = \sum_{i=1}^{n} [\beta^T X_i - \log{(\sum\nolimits_{j \epsilon R(t_i)}} \exp{(\beta^T X_j)}]$$

Maximizing this log-partial likelihood provides estimates of the regression coefficients β, which describe the influence of each predictor (e.g., age, educational level, risky sexual behaviour) on the risk of infection with Hepatitis B or HIV.

Robust Variance Estimator

To account for potential violations of the proportional hazards assumption and to provide more reliable estimates of the variance of the coefficients, the robust variance estimator (also known as the sandwich estimator) is employed. This method adjusts the standard errors of the coefficient estimates to be more resilient against certain types of model misspecifications, particularly when there are correlated events or when the proportional hazards assumption is not fully met.

The robust variance estimator is computed as follows:

$$Var(\hat{\beta}) = (X'WX)^{-1}X'WHWX(X'WX)^{-1}$$

where:

- X is the design matrix of covariates.
- W is a diagonal matrix of the weights, typically derived from the Cox model's estimated baseline hazard.
- H is the matrix of derivatives of the log-likelihood function with respect to the estimated coefficients.

Interpretation of Coefficients

The coefficients β from the Cox model are interpreted as log hazard ratios. For a one-unit increase in the covariate X_j the hazard ratio is:

$$Hazard\ Ratio = \exp(\beta_j)$$

Assumptions

Several assumptions must be met for the Cox regression model to be valid:

- **Proportional Hazards:** The hazard ratios are constant over time.

- **Independence:** Observations are independent of each other.
- **No Multicollinearity:** Covariates should not be highly correlated.
- **Linearity:** The effect of covariates is linear on the log hazard scale.

For your analysis, these assumptions should be carefully examined to ensure the reliability of the model when predicting the likelihood of infection in FSWs.

Application to Binary Outcomes

Though the Cox model is traditionally used for time-to-event data, it can be adapted to binary outcomes by fixing the time element and focusing on risk factors for infection. The event (infection) either occurs or does not, making the Cox model appropriate for examining how various risk factors (such as lack of age, condom use, number of sexual partners) influence the likelihood of infection among FSWs. The coefficients from this model can be used to estimate hazard ratios, providing insights into the relative risks of infection.
